# Preserved ability to blunt sympathetically‐mediated vasoconstriction in exercising skeletal muscle of young obese humans

**DOI:** 10.14814/phy2.14068

**Published:** 2019-04-29

**Authors:** Kanokwan Bunsawat, Georgios Grigoriadis, Elizabeth C. Schroeder, Alexander J. Rosenberg, Melissa M. Rader, Paul J. Fadel, Philip S. Clifford, Bo Fernhall, Tracy Baynard

**Affiliations:** ^1^ Integrative Physiology Laboratory Department of Kinesiology and Nutrition College of Applied Health Sciences University of Illinois at Chicago Chicago Illinois; ^2^ Department of Kinesiology College of Nursing and Health Innovation University of Texas at Arlington Arlington Texas

**Keywords:** Exercise blood flow, functional sympatholysis, obesity

## Abstract

Sympathetic vasoconstriction is attenuated in exercising muscles to assist in matching of blood flow with metabolic demand. This “functional sympatholysis” may be impaired in young obese individuals due to greater sympathetic activation and/or reduced local vasodilatory capacity of both small and large arteries, but this remains poorly understood. We tested the hypothesis that functional sympatholysis is impaired in obese individuals compared with normal‐weight counterparts. In 36 obese and normal‐weight young healthy adults (*n* = 18/group), we measured forearm blood flow and calculated forearm vascular conductance (FVC) responses to reflex increases in sympathetic nerve activity induced by lower body negative pressure (LBNP) at rest and during rhythmic handgrip exercise at 15% and 30% of the maximal voluntary contraction (MVC). FVC was normalized to lean forearm mass. In normal‐weight individuals, LBNP evoked a decrease in FVC (−16.1 ± 5.7%) in the resting forearm, and the reduction in FVC (15%MVC: −8.1 ± 3.3%; 30%MVC: −1.0 ± 4.0%) was blunted during exercise in an intensity‐dependent manner (*P* < 0.05). Similarly, in obese individuals, LBNP evoked a comparable decrease in FVC (−10.9 ± 5.7%) in the resting forearm, with the reduction in FVC (15%MVC: −9.7 ± 3.3%; 30%MVC: −0.3 ± 4.0%) also blunted during exercise in an intensity‐dependent manner (*P* < *0*.05). The magnitude of sympatholysis was similar between groups (*P* > 0.05) and was intensity‐dependent (*P* < 0.05). Our findings suggest that functional sympatholysis is not impaired in young obese individuals without overt cardiovascular diseases.

## Introduction

Obesity is an important cardiovascular disease risk factor associated with reduced physical activity levels, coupled with exercise intolerance (Vanhecke et al. 2009). While exercise is often recommended in part to combat obesity (Donnelly et al. 2009), our current understanding of how blood flow is controlled in order to meet the metabolic demands associated with exercise in obese individuals is incomplete. One potential mechanism underlying exercise intolerance in obesity is “functional sympatholysis” (Remensnyder et al. 1962; Tschakovsky et al. 2002). When functional sympatholysis is intact, locally released vasoactive substances within the exercising muscle can effectively attenuate sympathetically‐mediated vasoconstriction, contributing to the proper delivery of blood flow, oxygen, and nutrients to contracting skeletal muscles (Remensnyder et al. 1962; Tschakovsky et al. 2002; Hearon et al. 2016). Conversely, impaired functional sympatholysis contributes to maintained sympathetically‐mediated vasoconstriction and reduced perfusion in populations that exhibit sympathetic overactivation and diminished vascular function, such as in aging and hypertensive populations (Dinenno et al. 2005; Vongpatanasin et al. 2011; Saltin and Mortensen 2012).

Interestingly, while there is now evidence supporting obesity‐induced sympathetic overactivity (Alvarez et al. 2002; Gentile et al. 2007; Limberg et al. 2014), findings regarding sympathetically‐mediated vasoconstriction during exercise in obese individuals have been rather inconsistent, likely due, in part, to differences in study designs, age, and comorbidities (Negrao et al. 2001; Limberg et al. 2010, 2014; Thaning et al. 2011; Vongpatanasin et al. 2011). Furthermore, although data suggest progression of changes in the neurovascular control of blood flow with severe obesity (Limberg et al. 2016), literature involving younger obese participants without comorbidities is sparse (Agapitov et al. 2008; Limberg et al. 2012, 2014), and very little is known about the influence of obesity on functional sympatholysis. To our knowledge, Limberg et al. (2014) have been the only study to report on young obese individuals; however, these obese individuals had metabolic syndrome (MetS), which by definition is more than obesity alone. Thus, it still remains unclear whether young, otherwise healthy obese individuals would exhibit altered functional sympatholysis.

A better understanding of the neurovascular control of blood flow in obesity is a crucial step toward identifying therapeutic targets to mitigate the cardiovascular disease risks associated with obesity and to provide better healthcare. In this study, we sought to extend the findings by Limberg et al. (2014) and to test the hypothesis that young, otherwise healthy obese individuals (i.e., without MetS) would exhibit impaired functional sympatholysis compared with healthy normal‐weight controls. To test this hypothesis, we utilized lower body negative pressure (LBNP) to unload the cardiopulmonary baroreflex and induce a reflexive increase in sympathetic vasoconstriction at rest and during rhythmic handgrip exercise (Hansen et al. 1996). This LBNP stimulus has been used to evaluate functional sympatholysis in other populations (Fadel et al. 2004, 2012; Vongpatanasin et al. 2011).

## Methods

### Subjects

Thirty‐six young adults (18 normal‐weight and 18 obese) volunteered and completed a physical activity and health history questionnaire. Obese individuals had a body mass index (BMI) of 30–40 kg/m^2^. Exclusion criteria included any known cardiovascular, metabolic, renal, or respiratory disease. No participants were smokers or taking any cardiovascular medications, NSAIDS, or multivitamin/antioxidant supplements. Female participants had negative pregnancy tests and were studied during the early follicular phase of their menstrual cycle (*n* = 8), or during the placebo phase of oral contraceptives (*n* = 6). All participants were sedentary and were not engaged in regular aerobic exercise for the past 6 months (current physical activity level was <60 min/week based on a physical activity questionnaire). Written informed consent was obtained from all participants. All procedures were approved by the Institutional Review Board at the University of Illinois at Chicago and conformed to the guidelines set forth by the Declaration of Helsinki.

### Study design

Using a cross‐sectional design, all participants reported to the laboratory twice, at least 48 h apart, in the morning following an overnight fast (12 h) and were instructed to refrain from exercise, caffeine, and alcohol (24 h) before each visit. Day 1 involved baseline descriptive characteristics and experimental measurements, and Day 2 was the experimental day. For both visits, all vascular measures were obtained in the supine position following quiet rest (10–15 min) in a temperature‐controlled room (~22–24°C). On the experimental day (Day 2), participants underwent vascular and hemodynamic measurements at rest and during rhythmic handgrip exercise. Lower body negative pressure (LBNP) was applied as a sympathoexcitatory stimulus to examine functional sympatholysis. LBNP was applied both at rest and during rhythmic handgrip exercise.

### Day 1: experimental protocol and measurements

Upon arrival to the laboratory, all participants underwent anthropometric measurements, followed by measurements of seated resting blood pressure (BP). Then, all participants underwent a measurement of brachial artery flow‐mediated dilation (FMD) in the supine position, followed by a blood draw. Finally, all participants performed peak oxygen consumption (VO_2peak_) testing on a cycle ergometer.

#### Anthropometrics

Height, weight, and waist circumference were measured to the nearest tenth, and BMI was calculated (kg/m^2^). Body composition and lean forearm mass (using anatomical landmarks) were measured using whole‐body dual‐energy X‐ray absorptiometry (DEXA) (GE Lunar iDXA, GE Healthcare, Madison, WI), according to the manufacturer's guidelines.

#### Blood pressure (BP)

After resting quietly for 10 min, resting seated brachial BP of the nondominant arm was obtained using an automated oscillometric cuff (HEM‐907XL, Omron, Shimane, Japan). Measurements were made in duplicate, and the average BP value was used for analysis if the difference between the two values was ≤5 mmHg for both systolic and diastolic BP. Otherwise, a third measurement was obtained, and the closet two of the three values were averaged.

#### Brachial artery flow‐mediated dilation (FMD)

FMD was assessed using ultrasonography (Hitachi‐Aloka *α*‐7, Tokyo, Japan), as previously described (Kappus et al. 2017). Briefly, a rapid‐release cuff (DE Hokanson, Bellevue, WA) was placed below the elbow joint on the widest part of the forearm, with the arm being stabilized using an immobilizer cushion. The brachial artery was imaged in longitudinal sections using a high frequency (7.5 MHz) linear array probe. Dual mode was used to measure the arterial diameter (B‐mode) and Doppler velocity. The mean blood velocity (MBV) signals were corrected at an insonation angle of 60°. The sample volume was placed in the middle of the artery, with a large sampling area but care was taken not to extend beyond the vessel wall. Images were recorded at five frames per sec using Vascular Tools (MIA, Coralville, IA) during diastole only and analyzed offline using automated edge‐detection software (Brachial Analyzer, MIA, Coralville, IA). Baseline measures of resting brachial MBV and diameter were taken for 60 sec. The BP cuff was then inflated to 250 mmHg to induce ischemia for 5 min. Image capture was restarted 30 sec prior to cuff deflation and continued until 180‐sec post‐deflation. FMD (%) was calculated with the formula:

$$\text{FMD}\left(\%\right)=\frac{\text{Peak hyperemic diameter}\left(\text{cm}\right)-\text{Baseline diameter}\left(\text{cm}\right)}{\text{Baseline diameter}(\text{cm})}\times 100$$

Shear rate (sec^−1^) was calculated with the formula:

$$\text{Shear rate}\left(s^{-1}\right)=\frac{8\times \text{MBV}\left(\text{cm/s}\right)}{\text{Diameter}(\text{cm})}\times 100$$

Then, FMD (%) was normalized to shear stimulus using shear rate area under the curve (AUC) to the peak diameter.

#### Blood lipid profile

Venous blood samples were obtained in heparinized tubes and analyzed using Cholestech LDX (Cholestech Instruments, Hayward, CA) for the following variables: fasting plasma concentrations of low‐density lipoprotein cholesterol (LDL), high‐density lipoprotein cholesterol (HDL), total cholesterol, triglycerides, and glucose.

#### Peak oxygen consumption testing (VO_2peak_)

VO_2peak_ was measured using an open‐circuit spirometry metabolic system (TrueOne 2400, Parvo Medics, Sandy, UT) during an incremental graded cycling exercise test performed to exhaustion (Excaliber Sport, Lode, the Netherlands). The cycling exercise protocol was selected to support weight during locomotion especially in obese individuals. The participants began with a 1‐min warm‐up with no resistance. The first workload was set at 40 W and gradually increased by 30 W every 2 min until test termination. The participants pedaled at a cadence of 60–100 rpm. Ratings of perceived exertion were assessed once per stage. Following test termination, the recovery protocol began with 2‐min light cycling (0 W, 50 rpm), followed by 1 min of quiet sitting on the cycling ergometer. The test was terminated when participants met three of the following four criteria: (1) RPE score of 17 or greater on the Borg scale (scale 6–20), (2) respiratory exchange ratio of at least 1.1, (3) no change in HR with a change in workload, and/or (4) subjective volitional exhaustion.

### Day 2: experimental protocol and measurements

All participants were studied in the supine position. Beat‐to‐beat heart rate (HR) was recorded using an electrocardiogram (Biopac Systems, Santa Barbara, CA). Beat‐to‐beat BP was continuously recorded on the nondominant arm using finger photoplethysmography (Finometer Pro, Amsterdam, the Netherlands). Beat‐to‐beat HR and BP was recorded at a sampling rate of 1000 Hz (Biopac Systems, Santa Barbara, CA). Data were analyzed offline using WinCPRS (Absolute Aliens, Turku, Finland), and beat‐to‐beat BP was used to derive stroke volume (SV) and cardiac output (CO) using Modelflow software, which incorporates age, sex, weight, and height (Jansen et al. 1990, 2001; Kim et al. 2011), and then both were indexed to body surface area (stroke index (SI) and cardiac index (CI), respectively). These Modelflow‐derived data were reported as a change value in response to LBNP stimulation.

#### Rhythmic handgrip exercise

Maximal voluntary contraction (MVC) for each participant was selected as the greatest of at least three maximal squeezes of a handgrip dynamometer (TSD121C, Biopac Systems, Goleta, CA). All participants performed rhythmic handgrip to the rhythm of a metronome (20 handgrips per min; 50% duty cycle) at 15%, then 30% (EX15 and EX30) of MVC for 6 min, with a 10‐min rest between each exercise intensity. Force production was displayed on a projector screen to provide participants with visual feedback.

#### Reflex activation of sympathetic nerves

LBNP was used to produce reflex sympathetic vasoconstriction in the forearm. The participant's lower body was enclosed to the level of the iliac crest in the LBNP chamber. LBNP at −20 mmHg evokes reproducible reflex increases in muscle sympathetic nerve activity (Hansen et al. 1996; Fadel et al. 2004). LBNP at −20 mmHg was applied for 2 min at rest and during min 2‐4 of rhythmic handgrip exercise.

#### Forearm blood flow (FBF)

FBF was measured for a total of 6 min at rest and during rhythmic handgrip exercise using ultrasonography (Hitachi‐Aloka *α*‐7, Tokyo, Japan), and brachial diameter was analyzed offline as described above (under brachial FMD). MBV was analyzed using another commercially available blood velocity analysis software (Cardiovascular Suite, Quipu, Pisa, Italy) as previously described (Thomas et al. 2017). FBF was calculated with the formula:

$$\text{FBF}\left(\text{mL}\cdot {\text{min}}^{-1}\right)=\text{MBV}\left(\text{cm/s}\right)\times \pi \times {\left(\frac{\text{brachial diameter}(\text{cm})}{2}\right)}^{2}\times 60$$

Forearm vascular conductance (FVC, mL/min/mmHg) was calculated as follows:

$$\text{FVC}\left(\text{mL}\cdot {\text{min}}^{-1}{\left(100\;\text{mmHg}\right)}^{-1}\right)=\frac{\text{FBF}(\text{mL}\cdot {\text{min}}^{-1})}{\text{mean arterial pressure}(\text{mmHg})}\times 100$$

Then, FBF and FVC were normalized to forearm lean muscle mass (measured from DEXA) and annotated by nFBF and nFVC, respectively.

FBF, FVC, HR, and BP responses to LBNP were determined by calculating the difference between the average over the last 30 sec of baseline (or exercise) immediately preceding LBNP and the average of the last 30 sec during LBNP. Specifically, we utilized only the last 30 sec of exercise before LBNP for steady‐state blood flow (and also during LBNP) to ensure no effects of the onset kinetics. The main dependent variables were the relative change in FBF or FVC with LBNP (Δ%FBF and Δ%FVC) at rest and during exercise. These were calculated as follows:

$$\Delta \%\text{FBF}\;\text{or}\;\Delta \%\text{FVC}=\frac{\left(\text{FBF}\;\text{FVC}\;\text{LBNP}\right)-\left(\text{FBF or FVC before LBNP}\right)}{\text{FBF or FVC before LBNP}}\times 100$$

Finally, the magnitude of functional sympatholysis was calculated as the difference between the nFVC change to LBNP applied at rest [percent change (%Δ)] and the nFVC change in response to LBNP applied during exercise (%Δ).

This index of functional sympatholysis reflects the ability of muscle contractions to attenuate the reflex sympathetic vasoconstrictor response observed at rest (Dinenno et al. 2005; Wray et al. 2007).

### Statistical analysis

**Figure 1:**
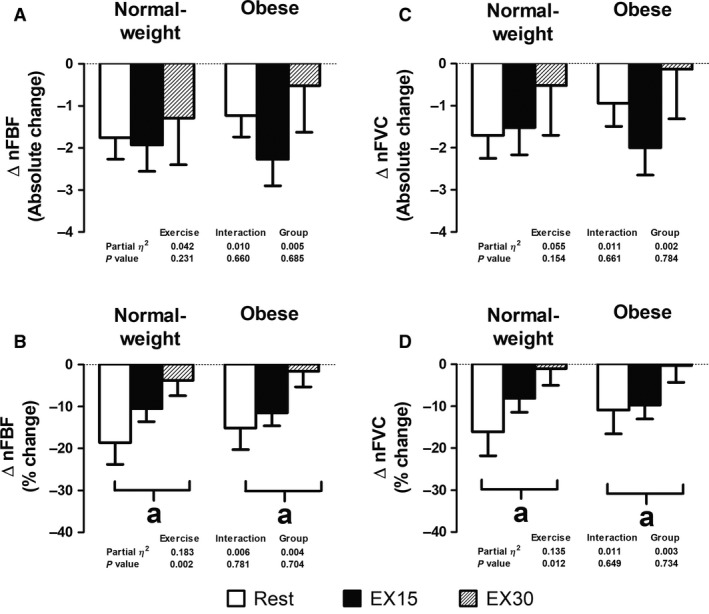
Absolute and percent (%) changes in normalized forearm blood flow (A–B) and calculated forearm vascular conductance (C–D) at rest and during rhythmic handgrip exercise at 15% (EX15) and 30% (EX30) of maximal voluntary contraction in response to lower body negative pressure stimulation were similar between groups. nFBF, forearm blood flow normalized to lean forearm mass; nFVC, forearm vascular conductance normalized to lean forearm mass. ^a^Time effect (*P* < 0.05). Data are mean ± SE.

**Figure 2:**
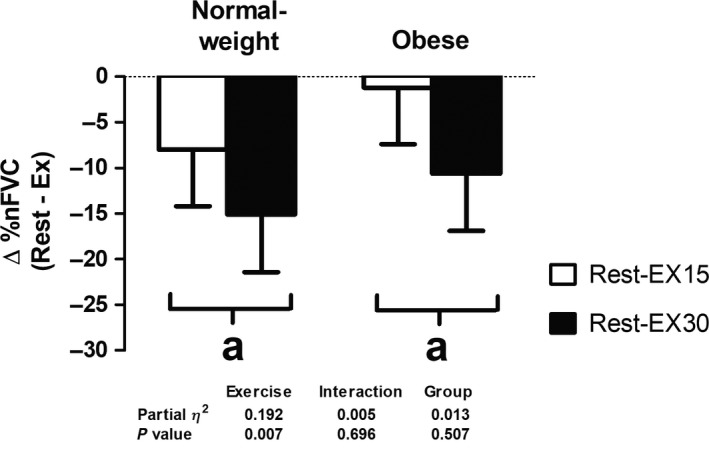
Magnitude of sympatholysis or the calculated difference in normalized forearm vascular conductance between rest and exercise at 15% (EX15) and 30% (EX30) of maximal voluntary contraction to lower body negative pressure stimulation was similar between groups. nFVC, forearm vascular conductance normalized to lean forearm mass. ^a^Time effect; Rest‐EX30 had greater changes than Rest‐EX15 (*P* < 0.05). Data are mean ± SE.

Data were checked for normality of distribution using Shapiro–Wilk tests, and non‐normally distributed data were normalized using natural log transformation. Descriptive characteristics, baseline differences, and percent change variables were compared between groups using an independent *t* test or nonparametric Mann–Whitney *U* test. The Mann–Whitney *U* test was utilized for data that could not be logged transformed. To test the responses to exercise only without LBNP (Tables 4 and 5), a 2 × 3 ANOVA with repeated measures [group (normal‐weight vs. obese) by time (rest, EX15, and EX30)] was conducted. To test the responses to LBNP at rest and during exercise (Tables 4 and 5), a 2 × 2 ANOVA with repeated measures [group (normal‐weight vs. obese) by time (before and during LBNP)] was conducted. To test the exercise intensity‐dependent responses, two separate analyses were conducted: (1) a 2 × 3 ANOVA with repeated measures [(group (normal‐weight vs. obese) by time (change values from LBNP at rest, EX15, and EX30)] (Fig. 1) and (2) a 2 × 2 ANOVA with repeated measures [(group (normal‐weight vs. obese) by time (rest‐EX15 and rest‐EX30)] (Fig. 2). Data are presented as mean ± SE. Alpha was set at *P* < 0.05. All data were analyzed using SPSS (V 21.0, IBM SPSS, Inc., Armonk, NY).

## Results

### Descriptive characteristics and flow‐mediated dilation

Obese individuals had higher weight, BMI, waist circumference, percent body fat, android fat (visceral and subcutaneous), android‐to‐gynoid ratio, and absolute VO_2peak_, as well as a lower VO_2peak_ (relative to body weight) compared with normal‐weight individuals (Tables 1 and 2, *P* < 0.05). However, no group differences were observed for any FMD variable (*P* > 0.05), except for a slightly higher peak blood velocity in the obese group (Table 3, *P* < 0.05). Statistically controlling for shear rate AUC, %FMD also remained similar between groups (Table 3, *P* > 0.05).

**Table 1:** Descriptive characteristics in normal‐weight (*n* = 18) and obese (*n* = 18) participants

	Normal‐weight	Obese
Age (years)	26 ± 1	27 ± 1
Sex (m/f)	11/7	11/7
Height (cm)	169.9 ± 1.3	172.2 ± 1.8
Weight (kg)	65.2 ± 1.6	96.5 ± 3.0^a^
BMI (kg/m^2^)	22.6 ± 0.4	32.4 ± 0.5^a^
Waist circumference (cm)	83.3 ± 1.4	109.4 ± 1.8^a^
Body fat (%)	29.9 ± 1.3	41.2 ± 1.3^a^
Android fat (g)	1424 ± 119	3485 ± 176^a^
Visceral fat (g)	346 ± 45	1015 ± 127^a^
Subcutaneous fat (g)	1078 ± 106	2471 ± 119^a^
Android‐to‐gynoid ratio	0.95 ± 0.05	1.20 ± 0.04^a^

Data are mean ± SE.

BMI, body mass index; SBP, systolic blood pressure; DBP, diastolic blood pressure; HDL, high‐density lipoprotein; LDL, low‐density lipoprotein; VO_2peak_, peak aerobic capacity.

a Different from normal‐weight participants (*P* < 0.05).

**Table 2:** Resting cardiovascular measures, blood measures, and peak oxygen uptake in normal‐weight (*n* = 18) and obese (*n* = 18) participants

	Normal‐weight	Obese
Seated SBP (mmHg)	111 ± 1	110 ± 3
Seated DBP (mmHg)	71 ± 1	74 ± 1
HR (bpm)	69 ± 2	68 ± 2
Total cholesterol (mg/dL)	177 ± 9	167 ± 8
HDL cholesterol (mg/dL)	59 ± 3	50 ± 3
LDL cholesterol (mg/dL)	103 ± 9	95 ± 8
Triglycerides (mg/dL)	94 ± 13	100 ± 12
Glucose (mg/dL)	95 ± 2	96 ± 3
VO_2peak_ (L/min)	2.07 ± 0.08	2.54 ± 0.12^a^
VO_2peak_ (mL/kg/min)	31.8 ± 1.0	26.2 ± 0.9^a^
VO_2peak_/FFM (mL/kg/min)	45.2 ± 1.6	44.3 ± 1.7

Data are mean ± SE.

SBP, systolic blood pressure; DBP, diastolic blood pressure; HDL, high‐density lipoprotein; LDL, low‐density lipoprotein; VO_2peak_, peak aerobic capacity.

a Different from normal‐weight participants (*P* < 0.05).

**Table 3:** Brachial artery flow‐mediated dilation variables in normal‐weight (*n* = 18) and obese (*n* = 18) participants

	Normal‐weight	Obese
Baseline brachial diameter (mm)	3.46 ± 0.09	3.68 ± 0.10
Baseline blood velocity (cm/sec)	14.2 ± 1.0	16.2 ± 1.0
Peak brachial diameter (mm)	3.78 ± 0.10	4.03 ± 0.11
%FMD	9.21 ± 0.29	9.66 ± 0.37
Blood velocity at peak diameter (cm/sec)	36.9 ± 3.9	37.6 ± 2.1
Shear rate at peak flow velocity (sec^−1^)	786 ± 85	744 ± 34
Peak blood velocity (cm/sec)	80.7 ± 3.8	91.3 ± 3.6^a^
Peak shear rate (sec^−1^)	1843 ± 109	1984 ± 105
Shear rate AUC (AU)	46,949 ± 4318	56,930 ± 4394
%FMD/Shear rate AUC (AU)	2.34 × 10^−4^ ± 0.28 × 10^−4^	1.89 × 10^−4^ ± 0.17 × 10^−4^

Data are mean ± SE.

FMD, flow‐mediated dilation; AUC, area under the curve; AU, arbitrary units.

a Different from normal‐weight participants (*P* < 0.05).

### Forearm blood flow responses to exercise

The exercise responses only (without LBNP) are shown in Tables 4 and 5. Brachial diameter increased with exercise in both groups (*P* < 0.05). At rest, normal‐weight individuals had lower FVC compared with obese individuals (Table 4, *P* < 0.05). However, when normalized to muscle mass, nFVC at rest was similar between groups (Table 4, *P* > 0.05). FBF, nFBF, FVC, and nFVC increased with exercise in an intensity‐dependent manner similarly in both groups (*P* < 0.05). HR also increased with exercise similarly in both groups (*P* < 0.05). MAP did not change from rest during exercise in either group (*P* > 0.05).

**Table 4:** Brachial artery diameter and blood flow responses to exercise with and without lower body negative pressure stimulation

	Group	Rest	Rest + LBNP	EX15	EX15 +LBNP	EX30	EX30 +LBNP
Diameter (mm)	Normal‐Weight	3.54 ± 0.13	3.47 ± 0.12^a^	3.56 ± 0.12	3.54 ± 0.11^a^	3.67 ± 0.12†‡	3.63 ± 0.12^a^
	Obese	3.73 ± 0.13	3.69 ± 0.12^a^	3.78 ± 0.12†	3.72 ± 0.11^a^	3.85 ± 0.12†	3.82 ± 0.11^a^
FBF (mL/min)	Normal‐Weight	72 ± 8	57 ± 8^a^	168 ± 19†	153 ± 19^a^	268 ± 31†‡	261 ± 34
	Obese	88 ± 8	74 ± 8^a^	206 ± 19†	181 ± 19^a^	306 ± 31†‡	298 ± 34
nFBF (AU)	Normal‐Weight	7.8 ± 0.8	6.0 ± 0.7^a^	17.5 ± 1.3†	15.5 ± 1.3^a^	27.6 ± 2.0†‡	26.3 ± 2.1
	Obese	7.7 ± 0.8	6.5 ± 0.7^a^	17.9 ± 1.3†	15.7 ± 1.1^a^	26.4 ± 2.0†‡	25.9 ± 2.1
FVC (mL/min/mmHg)	Normal‐Weight	74 ± 9	60 ± 10 a	165 ± 18†	154 ± 18^a^	260 ± 26†‡	260 ± 29
	Obese	88 ± 9^b^	78 ± 9^a^	206 ± 18†	185 ± 18^a^	290 ± 26†‡	287 ± 29
nFVC (AU)	Normal‐Weight	7.9 ± 0.9	6.2 ± 0.8^a^	17.2 ± 1.4†	15.7 ± 1.2^a^	27.0 ± 1.8†‡	26.5 ± 2.0
	Obese	7.8 ± 0.9	6.8 ± 0.8^a^	18.0 ± 1.4†	16.0 ± 1.2^a^	25.1 ± 1.8†‡	25.0 ± 2.0

Data are mean ± SE.

LBNP, lower body negative pressure; EX15, exercise at 15% of the maximal voluntary contraction; EX30, exercise at 30% of the maximal voluntary contraction; FBF, forearm blood flow; nFBF, forearm blood flow normalized to lean forearm mass; FVC, forearm vascular conductance; nFVC, forearm vascular conductance normalized to lean forearm mass; AU, arbitrary units.

a Different than before LBNP stimulation (*P* < 0.05).

b Different from normal‐weight participants at this time point (*P* < 0.05).

†Different than rest; ^‡^Different than EX15 (Exercise Only, Without LBNP) (*P* < 0.05).

**Table 5:** Hemodynamic responses to exercise with and without lower body negative pressure stimulation

	Group	Rest	Rest +LBNP	EX15	EX15 +LBNP	EX30	EX30 +LBNP
MAP (mmHg)	Normal‐weight	101 ± 2	98 ± 2^a^	102 ± 2	99 ± 2^a^	102 ± 2	100 ± 2^a^
	Obese	101 ± 2	96 ± 2^a^	100 ± 2	98 ± 2^a^	105 ± 2	103 ± 2^a^
HR (bpm)	Normal‐weight	61 ± 2	62 ± 2	65 ± 2^b^	67 ± 2^a^	66 ± 2^b^^‡^	69 ± 2^a^
	Obese	61 ± 2	61 ± 2	63 ± 2^b^	67 ± 2^a^	66 ± 2^b^^‡^	70 ± 2^a^
SV (mL)	Normal‐weight	Δ −9 ± 2		Δ −11 ± 2		Δ −10 ± 2	
	Obese	Δ −10 ± 2		Δ −11 ± 2		Δ −11 ± 2	
SI (mL/m^2^)	Normal‐weight	Δ −5 ± 1		Δ −6 ± 1		Δ −6 ± 1	
	Obese	Δ −5 ± 1		Δ −5 ± 1		Δ −5 ± 1	
CO (L/min)	Normal‐weight	Δ −0.4 ± 0.1		Δ −0.6 ± 0.2		Δ −0.5 ± 0.2	
	Obese	Δ −0.7 ± 0.1		Δ −0.4 ± 0.2		Δ −0.3 ± 0.2	
CI (L/min/m^2^)	Normal‐weight	Δ −0.3 ± 0.1		Δ −0.4 ± 0.1		Δ −0.3 ± 0.1	
	Obese	Δ −0.3 ± 0.1		Δ −0.2 ± 0.1		Δ −0.1 ± 0.1	

Data are mean ± SE.

MAP, mean arterial pressure; HR, heart rate; SV, stroke volume; SI, stroke index or stroke volume normalized to body surface area; CO, cardiac output; CI, cardiac index or cardiac output normalized to body surface area. SV, SI, CO, and CI were reported as change values in response to LBNP stimulation.

a Different than before LBNP stimulation (*P* < 0.05).

Different from normal‐weight participants (*P* < 0.05).

b Different than rest; ^‡^Different than EX15 (Exercise Only, Without LBNP) (*P* < 0.05).

### Forearm blood flow and forearm vascular conductance responses to LBNP at rest and during exercise

Brachial artery responses to LBNP at rest and during exercise are shown in Table 4, and Figures 1 and 2. In normal‐weight individuals, LBNP stimulation reduced FBF, nFBF, FVC, and nFVC at rest and at EX15, but not EX30, with these reductions being attenuated during exercise in an intensity‐dependent manner (Table 4 and Fig. 1). Specifically, LBNP reduced nFVC by ‐16.1 ± 5.7% at rest, but only by ‐8.1 ± 3.3% at EX15, and by ‐1.0 ± 4.0% at EX30 in normal‐weight individuals (Fig. 1, *P* < 0.05). Similarly, obese individuals also exhibited comparable reductions in FBF, nFBF, FVC, and nFVC in response to LBNP stimulation at rest and at EX15, but not EX30, with these reductions also being attenuated during exercise in an intensity‐dependent manner (Table 4 and Fig. 1). Specifically, LBNP reduced nFVC by −10.9 ± 5.7% at rest, but only by −9.7 ± 3.3% at EX15, and by −0.3 ± 4.0% at EX30 in obese individuals (Fig. 1, *P* < 0.05). No group differences in nFBF or nFVC responses to LBNP were observed at rest or during exercise at both intensities (Table 4 and Fig. 1). In addition, no group differences at rest or in response to LBNP were found for brachial artery diameter (*P* > 0.05, Table 4). However, LBNP elicited slight but significant reductions in brachial artery diameter during both exercise intensities similarly in both groups (*P* < 0.05, Table 4).

### Magnitude of sympatholysis

In both groups, the percent reductions in FVC and nFVC in response to LBNP stimulation became more attenuated with increasing exercise intensity (Fig. 1, *P* < 0.05). To further quantify these changes, we calculated the magnitude of sympatholysis, that is, the difference in nFVC between rest and exercise (Fig. 2) (Dinenno et al. 2005; Wray et al. 2007). The magnitude of sympatholysis was similar between groups and was intensity‐dependent, with a greater sympatholysis at EX30 versus EX15 (Fig. 2, *P* < 0.05).

### Hemodynamic responses to LBNP at rest and during exercise

Hemodynamic responses to LBNP are depicted in Table 5. LBNP stimulation reduced MAP slightly but significantly at rest and during rhythmic handgrip exercise at EX15 and EX30 similarly in both groups (*P* < 0.05). LBNP stimulation also reduced SV, SI, CO, and CI, as expected, at rest and during rhythmic handgrip exercise at both exercise intensities similarly in both groups. LBNP stimulation increased HR slightly in both groups at EX15 and EX30 (*P* < 0.05), but not at rest (*P* > 0.05).

## Discussion

The major findings of the present study are twofold. First, a similar sympathetic vasoconstriction in response to LBNP at rest was observed in young, otherwise healthy obese individuals compared with healthy normal‐weight controls. Second, during rhythmic handgrip exercise, sympathetic vasoconstriction was attenuated in the exercising forearm muscles of young, otherwise healthy obese individuals in an intensity‐dependent manner, and the magnitude of sympatholysis was similar to healthy normal‐weight controls. Thus, in contrast to our hypothesis, our findings demonstrate that during small muscle mass contractions, functional sympatholysis was preserved in young, otherwise healthy obese individuals (without MetS). Furthermore, our findings of preserved functional sympatholysis in obesity alone extend the findings of Limberg et al. (2014) in MetS subjects and provide an additional important understanding of the neurovascular control of blood flow in human obesity.

### Sympathetic vasoconstriction at rest

The LBNP stimulus evokes sympathetically‐mediated vasoconstriction by cardiopulmonary baroreflex unloading, which evokes a global endogenous release of norepinephrine from sympathetic nerve endings and subsequent binding to *α*_1_/*α*_2_‐adrenergic receptors (Rongen et al. 1996). Using this method, we observed similar reductions in FBF and FVC (both absolute and relative values) in response to LBNP stimulation at rest in young obese compared with normal‐weight individuals (Table 1 and Fig. 1), suggesting that obesity alone did not enhance sympathetically‐mediated vasoconstriction at rest in young, otherwise obese individuals. Our finding is in contrast to a previous report of exaggerated sympathetic vasoconstriction in obese individuals who were slightly older than our cohort (~3 years) (Kuniyoshi et al. 2003). However, we measured FBF using Doppler ultrasound during sympathetic stimulation with LBNP, whereas the previous study measured FBF using venous occlusion plethysmography during a cold pressor test (Kuniyoshi et al. 2003), which induces endogenous release of norepinephrine upon a sudden and increasingly painful cold stress (Silverthorn and Michael 2013). Thus, these differing approaches may have contributed to these discrepant findings.

Limberg et al. (2012, 2014) also evaluated sympathetically‐mediated vasoconstriction in young obese individuals; however, their obese participants had MetS and sympathetically‐mediated vasoconstriction was interrogated directly in the exercising forearm using an intra‐arterial infusion of specific *α*_1_‐ and *α*_2_‐adrenergic receptor agonists. Using this method, the magnitude of reductions in FVC in response to intra‐arterial infusion of an *α*_1_‐adrenergic agonist was numerically greater in young obese individuals with MetS (~33%) compared with normal‐weight controls (~25%), demonstrating a trend for increased sympathetic vasoconstrictor responsiveness (Limberg et al. 2012, 2014). When challenged with an *α*_2_‐adrenergic agonist, FVC was also reduced more in MetS subjects (~61%) compared to normal‐weight controls (~42%) (Limberg et al. 2012, 2014). While our findings are not similar to those of Limberg et al. (2012, 2014), it is important to appreciate the heterogeneity and complexity of obesity in terms of neurovascular control of blood flow owing to different severities and rates of progression.

### Sympathetic vasoconstriction during exercise

During exercise alone, we observed similar intensity‐dependent increases in FBF and FVC (both absolute and normalized values) in both groups, which is consistent with previous findings (Limberg et al. 2010). However, the exercise responses alone, without an additional cardiovascular challenge, may not be sufficient to unmask any presence of impaired functional sympatholysis associated with obesity alone. To date, Limberg et al. (2014) is the only study to evaluate functional sympatholysis in young obese individuals and found impaired functional sympatholysis in response to an intra‐arterial infusion of an *α*_2_‐adrenergic receptor agonist, but preserved functional sympatholysis in response to an intra‐arterial infusion of an *α*_1_‐adrenergic receptor agonist during rhythmic handgrip exercise at 15% of the MVC (Limberg et al. 2014). However, as mentioned previously, these obese individuals had MetS, which by definition is more than obesity alone. Functional sympatholysis was also evaluated pharmacologically during separate stimulation of *α*_1_‐ or *α*_2_‐adrenoreceptors, instead of combined *α*‐adrenergic receptor stimulation (Limberg et al. 2014). Thus, in an attempt to extend findings of Limberg et al. (2014), we evaluated whether obesity alone would impair functional sympatholysis during a global increase in sympathetically‐mediated vasoconstriction. In contrast to our hypothesis, we found that young, otherwise healthy obese individuals exhibited preserved functional sympatholysis and that the ability to blunt reflex sympathetic vasoconstriction during small muscle mass contractions at both 15% and 30% of the MVC was comparable between young obese and normal‐weight individuals (Figs. 1 and 2). Our findings of an exercise intensity dependent functional sympatholysis are consistent with previous work using LBNP, cold pressor test, or infusion of tyramine in young normal‐weight individuals (Hansen et al. 2000; Tschakovsky et al. 2002; Dinenno et al. 2005; Watanabe et al. 2007; Wray et al. 2007).

It is possible that the preserved functional sympatholysis in our obese individuals was because they were relatively young and healthy and without overt cardiovascular disease, such as hypertension (Vongpatanasin et al. 2011), which has been implicated in impaired functional sympatholysis. In line with this, one study using LBNP stimulation (also at ‐20 mmHg) reported impaired functional sympatholysis in the exercising forearm (30% of MVC) of obese individuals with hypertension (~47 years of age; BMI ~30 kg/m^2^) compared with age‐matched, overweight normotensive individuals (BMI ~29 kg/m^2^), and that the impairment was attributed to an angiotensin‐dependent mechanism (Vongpatanasin et al. 2011). Once again, the comparison to the present results suggests that obesity alone does not impair functional sympatholysis. In support of our findings, work from Thaning et al. (2011) also reports that exercise‐induced increases in leg blood flow and leg vascular conductance were maintained in middle‐aged overweight individuals with type 2 diabetes (~55 years of age, BMI 29.1 kg/m^2^), even during increased sympathetic vasoconstriction induced by tyramine infusion. Moreover, the vasodilatory response to acetylcholine was also intact in overweight individuals with type 2 diabetes compared with age‐matched healthy controls (~55 years of age, BMI 26.5 kg/m^2^), suggesting preserved endothelial function occurs concomitantly with “normal” functional sympatholysis (Thaning et al. 2011).

Recent work from Hearon et al. (2016) highlights the role of endothelium‐dependent signaling during muscle contraction in blunting sympathetic vasoconstriction in humans. We also assessed brachial artery FMD and reported no group differences in endothelial function (%FMD) and a slightly higher peak blood velocity during cuff release in the obese cohort, suggesting healthy macro‐ and microvascular endothelial function in our young obese cohort. As such, our data and that of Thaning et al. (2011) indicate that impaired functional sympatholysis may occur only in the presence of endothelial dysfunction, likely due to an imbalance of vasodilators and vasoconstrictors (Rajendran et al. 2013). However, given inherent differences in vasodilator responses between the arms and the legs (Newcomer et al. 2004), as well as differences between populations (young otherwise healthy obese vs. middle‐aged overweight type 2 diabetics), we cannot make direct comparisons between these findings (Thaning et al. 2011). Interestingly, higher fitness levels have been demonstrated to potentially produce a differential pattern of functional sympatholysis that is dependent on exercise training status (Wray et al. 2007). Although VO_2peak_ was slightly lower in the obese group when normalized to body weight, their VO_2peak_ per fat‐free mass was similar. Furthermore, VO_2peak_ data were not correlated to any measures of sympatholysis or exercise blood flow in the present study (data not shown), suggesting that our findings were unrelated to fitness levels in the present study.

### Experimental considerations and limitations

First, our exercise protocol engaged a small muscle mass in the arm, and it remains unclear if obesity would impact the response during exercise involving a larger muscle mass, but a previous study suggests that muscle mass does not influence functional sympatholysis (Wray et al. 2004). Second, sex differences may exist in the responses to our protocol (Gotshall 2000; Kneale et al. 2000; Hart et al. 2009); however, our study was not originally powered to detect sex differences, and when we performed a subanalysis for sex differences, our *P*‐values were somewhat large and ranged between 0.26 and 0.30 for the percent change in nFBF and nFVC. Obviously, this is an important area and warrants future examination. Third, we did not have measures of MSNA, which would provide more complete information regarding the neurovascular control of blood flow. This microneurographic technique was attempted at the radial nerve, although in many subjects the MSNA signal was lost over time due to subject movement. However, both groups exhibited comparable reductions in resting FVC during LBNP stimulation, suggesting sympathetically‐mediated vasoconstriction was similar in both groups.

## Conclusions

In conclusion, young, otherwise healthy obese individuals maintain the ability to attenuate reflex sympathetic vasoconstriction during rhythmic handgrip exercise compared with age‐matched normal‐weight counterparts.

## Conflict of Interest

There are no conflict of interest.
